# The association of *NR1H3* gene with lipid deposition in the pig

**DOI:** 10.1186/s12944-016-0269-5

**Published:** 2016-05-26

**Authors:** Bo Zhang, Peng Shang, Yangzong Qiangba, Aishi Xu, Zhixiu Wang, Hao Zhang

**Affiliations:** National Engineering Laboratory for Animal Breeding, China Agricultural University, No. 2 Yuanmingyuan West Rd., Beijing, 100193 People’s Republic of China; College of Agriculture and Animal Husbandry, Tibet University, Linzhi, 860000 People’s Republic of China

**Keywords:** *NR1H3*, Lipid deposition, SNP, Pig

## Abstract

**Background:**

Nuclear receptor subfamily 1, group H, member 3 (*NR1H3*, an alias for Liver X receptor α, LXRα) is a member of the LXR nuclear receptor super family and is an important regulator of lipid and fatty acid accumulation in the liver, adipose and skeletal muscle.

**Methods:**

In this study, single-nucleotide polymorphisms (SNPs) from six populations of pig (*Sus scrofa*) were screened by PCR-sequencing and genotyped, and its association with backfat thickness was analyzed in a population of New Huai line (NHP, *n* = 117). In addition, quantitative real-time PCR and western blot were used to measure expression of *NR1H3* in the liver tissue, backfat and *longissimus dorsi* muscle of DSP (*n* = 10), TP (*n* = 10) and YY (*n* = 10) pigs.

**Results:**

Three SNPs (exon2-C105T, exon2-G106C, and exon5-A201C) were screened and exon5-A201C was identified; the genotype frequencies were significantly different between indigenous and introduced breeds. The CC genotype was associated with higher backfat thickness than the AA and AC genotypes in the NYP. *NR1H3* mRNA and protein expression were higher in the liver and *longissimus dorsi* of DSP and TP than in those of YY. This increased *NR1H3* expression might be associated with higher lipid deposition. *NR1H3* expression in the backfat of YY was not lower than that in DSP or TP, which might because *NR1H3* has an alternative regulatory function for lipid metabolism in the subcutaneous fat of pigs.

**Conclusions:**

Our results suggest that allele A of the exon5-A201C in *NR1H3* may promote a reduction in backfat thickness, and differences in *NR1H3* expression may be associated with differences in lipid deposition capacity among pigs.

## Background

*NR1H3* is a member of the *LXR* nuclear super receptor family [1]. These genes are mainly distributed in the liver, adipose tissue, kidney, small intestine, and macrophages, and their main physiological function is to maintain the homeostasis of cholesterol level, lipoprotein metabolism, and fat synthesis [2–4].

*NR1H3* is activated by oxysterols and intermediates from the cholesterol biosynthetic pathway. *NR1H3* signaling is associated with the development of pathogenic conditions, such as hepatic steatosis. *NR1H3* signaling contributes to increases in hepatic triglyceride content by upregulating lipogenic genes. Downregulation of *NR1H3* expression in steatotic hepatocytes in vitro decreases the triglyceride content of the hepatocytes and promotes the recovery from hepatocyte steatosis [5]. Genetic studies have defined the *NR1H3* subtype as the major regulator of hepatic lipogenesis [6, 7].

Pig *NR1H3* maps to chromosome 2, is comprised of ten exons, and is 1711 bp in length. Based on the important roles of *NR1H3* in lipogenesis and myogenesis in humans and mice [8, 9], as well as their location in the pig genome, *NR1H3* is an attractive candidate gene for lean muscle growth and fat content, and might influence carcass composition and meat quality in pigs. The aims of this study were to investigate differences in *NR1H3* expression and SNP among several breeds of pig, to analyze the association of *NR1H3* with lipid deposition in pigs, and to provide a theoretical basis for the use of molecular markers in marker assisted selection (MAS).

## Methods

### Experimental materials

A total of 330 ear tissue samples were collected from six populations of pig (*Sus scrofa*) for DNA extraction. The populations included Tibetan pigs (TP, *n* = 50) from Linzhi, Tibet; Diannan small-eared pigs (DSP, *n* = 59) from Xishuang Banna, Yunnan; Landrace pigs (LP, *n* = 54) from Beijing Zhongshun Jingsheng Farm; Dapulian black pigs (DBP, *n* = 42) from Jining, Shandong; and Yorkshire (YY, *n* = 56); Duroc pigs (DP, *n* = 69) from Hefei, Anhui of China.

In addition, three groups of pigs (DSP, TP, and YY) were raised under the same conditions in Beijing. Ten individuals from each group were humanely slaughtered at 6 months of age using a normal procedure. Tissue samples of liver, backfat adipose and *longissimus dorsi* muscle were collected from each individual and immediately frozen in liquid nitrogen, and then stored at −80 °C until RNA and protein extraction.

All procedures were carried out in strict accordance with the protocol approved by the Animal Welfare Committee of China Agricultural University (Permit Number: XK622).

### DNA, RNA, protein extraction, and cDNA preparation

Genomic DNA was isolated from the ear tissues using the method described by Sambrook et al. [10], dissolved in Tris-EDTA (TE) buffer, and stored at −20 °C.

Total RNA was extracted from tissues with TRIZOL® Reagent (Invitrogen, San Diego, CA, USA) according to the manufacturer’s instructions. The concentration and purity of the RNA samples were checked using a Nanodrop 2000 Biophotometer (Thermo Fisher Scientific Inc., West Palm Beach, FL, USA; pure RNA samples were indicated by a 260/280 nm absorbance ratio of 1.8–2.0) and electrophoresed to verify their integrity. Two-microgram RNA samples in a 20 μl reaction volume were reverse transcribed to cDNA using ImProm-IITM Reverse Transcriptase (Promega Biotech Co., Ltd., China).

Total protein was isolated from the liver, backfat and *longissimus dorsi* muscle using SDS Lysis Buffer (P0013B, Beyotime Ltd. China). Protein content was measured using an enhanced BCA protein assay kit (P0010, Beyotime, Ltd. China).

### SNP screening

**Fig. 1:**
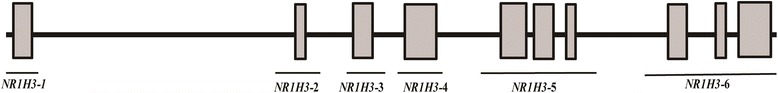
Structure of the pig *NR1H3* gene and the positions of primers used for SNP identification. The thick black lines represent introns; the grey blocks represent exons of the *NR1H3* gene; the thin black lines represent positions of amplicons. Pig total DNA was used as PCR templates for the *NR1H3-1*, *NR1H3-2*, *NR1H3-3*, *NR1H3-4*, *NR1H3-5* and *NR1H3-6* primers

Six pairs of primers for pig *NR1H3* (NM_001101814) were designed using Primer Premier 5.0 software (PREMIER Biosoft International, CA, USA). Amplicons of the primers covered all exons of the gene (Table 1 and Fig. 1). PCR products amplified from 10 samples of each group were pooled and sequenced to identify SNPs using Chromas Pro v.1.33 (Technelysium Pty Ltd, Helensvale, Australia) and DNAMAN6.0 software (Lynnon, Pointe-Claire, QC, Canada).

**Table 1:** Six pairs of primers used for SNP identification in pig *NR1H3*

Name	Sequences (5′ to 3′)	Amplicon region	Amplicon size (bp)	annealing temperature
*NR1H3-*1	F: TCCCACTCTGAGGTTCTTTTT	Exon 1	235	58 °C
	R: CTTACGGACCTGACACTGGA			
*NR1H3-*2	F:GCCAGGAAAGCCTTAGCACA	Exon 2	361	60 °C
	R: AGGAGGCAAGCAACAGCAAG			
*NR1H3-*3	F:CTGAGACCCCCCCTGTGC	Exon 3	259	59 °C
	R: GCCCCTACCTCCTCCAAATC			
*NR1H3-*4	F: GAACATTAAGCCTCTTCCAT	Exon 4	347	54 °C
	R: TTCCCTCTTTCCTATCAGC			
*NR1H3-*5	F: ATCTCTTCCTTGTCTTTACCC	Exon 5, exon 6 and exon 7	889	56 °C
	R: CAATCCCTTTGTGATCTCAG			
*NR1H3-*6	F: AGCAGTTTCCTCAGTTGAGC	Exon 8, exon 9 and exon 10	1029	56 °C
	R: AGGGTCAGTACCGTCTTCAC

### SNP genotyping

After screening, the SNP genotypes of exon 5-A201C of the *NR1H3* gene were determined using PCR-restriction fragment length polymorphism (RFLP). The primers for *NR1H3* exon 5 were forward-AAG AAA CTG AAG CGG CAA GAG and reverse-ATC GCA GAG GTC TTT AGG AGG, and the restriction enzyme was *Taq* I (New England Biolabs, USA). The amplicon size was 426 bp, and individuals with 348 and 113 bp fragments had genotype AA; individuals with 348, 290, 113, and 58 bp fragments had genotype AC; and individuals with 290, 113, and 58 bp fragments had genotype CC.

### Association of *NR1H3* genotype with backfat thickness

We collected ear tissue samples from 117 individuals of the New Huai line (NHP) from Anhui Kexin Farm. Backfat thickness for each animal was adjusted to 90 kg body-weight using a previously described equation [11]. The exon 5-A201C genotypes for each animal were determined using PCR- *Taq* I-RFLP, and the phenotypic differences among the three genotypes were analyzed using the PROC GLM procedure in SAS 9.1 (SAS Institute, Inc.).

### Quantitative PCR

*NR1H3* mRNA expression in liver tissue, backfat adipose and *longissimus dorsi* muscle of ten biological replicates were measured using real-time PCR (RT-PCR). RT-PCR was conducted in triplicate in a Bio-Rad CFX96 System (Bio-Rad, USA) with 1.0 μL cDNA, 0.5 μL of the respective forward and reverse primers (10.0 nmol/μL) and 10.0 μL SYBR Green qPCR SuperMix (Transgen, Beijing, China), for a total volume of 20 μL. The RT-PCR basic protocol included an initial denaturation step (95 °C for 3 min) followed by 40 cycles of 95 °C for 10 s and 60 °C for 20 s, and a final melting curve analysis. The amplification efficiency was calculated as 10^-1/slope^ of the standard curve derived from 5 standards created using a 5-fold dilution series of cDNA. The expression values were normalized using glyceraldehyde-3-phosphate dehydrogenase (*GAPDH*) expression as the internal reference gene to calculate ddCt expression values. Sets of primers designed with the Primer Premier 5.0 software were as follows: *NR1H3* (NM_001101814) (forward: CCT AAA GAC CTC TGC GAT TGA, reverse: GGT TGA TGA ACT CCA CCT GC); *GAPDH* (NM_001206359) (forward: GGT CAC CAG GGC TGC TTT TA; reverse: CCT TGA CTG TGC CGT GGA AT).

### Western blotting

*NR1H3* protein expression in the liver tissue, backfat adipose and *longissimus dorsi* muscle of ten biological replicates were measured using western blotting. About 30 mg of tissue was homogenized in lysis buffer (10 mmoL/L NaH_2_PO_4_, 1 mmoL/L EDTA, 10 mmoL/L β-mercaptoethanol, 0.25 % Triton X-100 and 0.02 % NaN_3_, adjusted to pH 6.8) using a Mixer Mill MM400 (Retsch, Germany) for 5 min, and then centrifuged at 10,000 × *g* for 10 min at 4 °C. Protein concentrations were determined using a Protein Assay Kit (Bio-Rad). Proteins (40 μg) were separated by sodium dodecyl sulfate polyacrylamide gel electrophoresis (SDS-PAGE) according to manufacturer’s instructions (BioRad). The Separated proteins were transferred to Immobilon-P Transfer Membranes (IPVH00010) for 2 h at 300 mA using a Bio-Rad Criterion Blotter. Membranes were blocked overnight in a blocking buffer (P0023B, Beyotime Ltd., China), and then incubated at 4 °C for 2 h with primary mouse monoclonal *GAPDH* (AG019, Beyotime Ltd., China) and *NR1H3* antibodies (ab82774, Abcam, UK). Both *GAPDH* and *NR1H3* were diluted in primary antibody dilution buffer (1:1000 dilution, P0023A, Beyotime Ltd., China). After three washes with PBST (phosphate buffer saline containing 0.1 % Tween 20), membranes were incubated for 1 h with secondary HRP-labeled goat anti-mouse IgG antibody (H + L, A0216, Beyotime Ltd., China) diluted in secondary antibody dilution buffer (1:1000 dilution, P0023D, Beyotime Ltd., China). Immune complexes were visualized using an eECL Western Blot Kit (CW0049A, CWBIO Ltd., China), according to the manufacturer’s directions. The protein blots were analyzed using Image J 1.44 software (NIH, USA) to determine the expression ratios of *NR1H3* and *GAPDH*.

### Statistical analysis

Expression levels were analyzed by one-way ANOVA using SAS 9.1 Software (SAS Inst. Inc., Cary, NC). Graphs were prepared using SigmaPlot 10.0 (Systat Software, San Jose, CA) and data are presented as mean ± standard error. *χ*^2^ tests were used to analyze the distribution of genotypes and differences in allele frequencies between populations.

## Results

### SNP identification

**Fig. 2:**
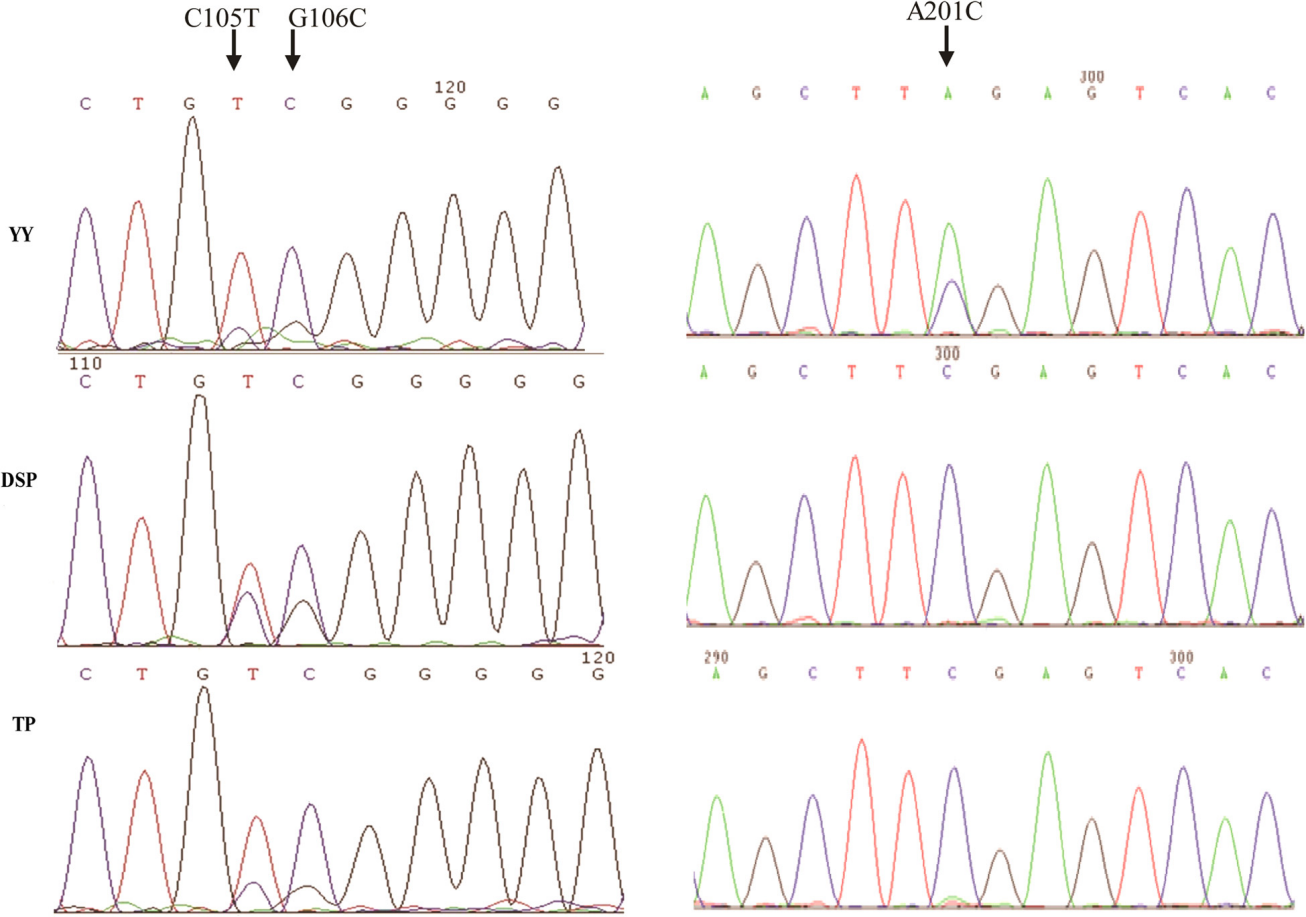
Sequencing chromatograms of SNPs of exon2-C105T, exon2-G106C, and exon5-A201C. Note: YY, Yorkshire pig; DSP, Diannan small-eared pig, TP, Tibetan pig. The arrows indicate the SNP site

The PCR amplicons obtain using the primers listed in Table 1 covered all 10 exons of the *NR1H3* gene full coding regions using (Fig. 1). Sanger sequencing revealed 3 SNPs in the full-coding regions of *NR1H3*: exon2-C105T, exon2-G106C, and exon5-A201C in the full-coding regions of the *NR1H3* gene (Fig. 2). Sequencing chromatograms (Fig. 2) showed that the sites of exon5-201 were all C in the DSP and TP populations, but were A/C in the YY population. Therefore, the SNP of exon5-A201C is genotyped using PCR-RFLP in more individuals and more populations.

### SNP genotype frequency

**Fig. 3:**
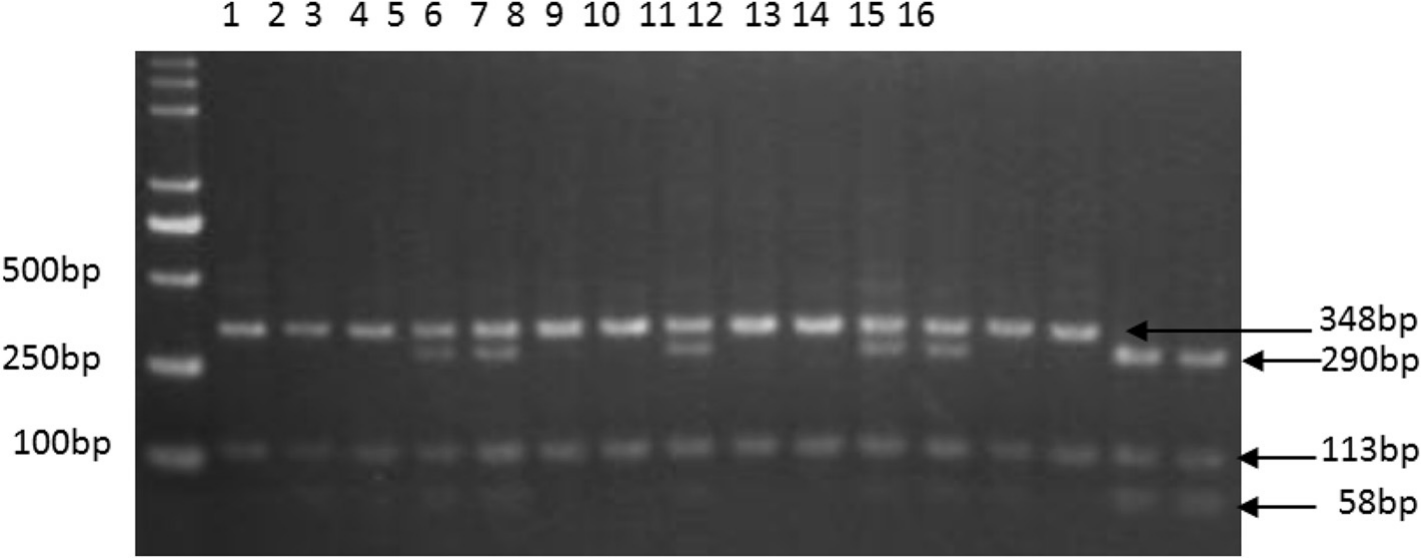
The electrophoresis of PCR-*Taq* I-RFLP for *NR1H3* exon 5-A201C in pigs. Note: The individual with 348 and 113 bp fragments had genotype AA (lanes 1, 2, 3, 6, 7, 9, 10, 13, 14), the individual with 348, 290, 113, and 58 bp fragments had genotype AC (lanes 4, 5, 8, 11, 12), and the individual with 290, 113, and 58 bp fragments had genotype CC (lanes 15, 16)

As seen from the PCR-*Taq* I-RFLP electrophoresis of the *NR1H3* exon 5-A201C, individuals had 348,290, 113 and 58 bp fragments (Fig. 3). The genotypes and allele frequencies of exon 5-A201C in six pig breeds are shown in Table 2. The CC and AC genotypes were detected in TP and DSP, the AA and AC genotypes were found in YY and DP, and three genotypes (AA, AC, and CC) were found in DBP and LP. The AA genotype predominated in the introduced lean-type breeds (YY, LP, DP), whereas the CC genotype predominated in the Chinese indigenous fat-type breeds (TP, DSP). Only the DBP breed was predominantly AC genotype. The A allele frequency in the lean-type breeds was significantly higher than in the fat-type breeds (*P* < 0.01). Hardy-Weinberg equilibrium tests showed that the frequency distribution of the genotypes in these populations reached equilibrium (*P* > 0.05). Chi-square tests showed that the genotype distribution was significantly different between the lean- and fat-type breeds (*P* < 0.05).

**Table 2:** The genotype and allele frequencies of exon 5 A201C in six pig breeds

Breed/Total number	Genotype frequency(number/frequency)				Allele gene frequency	
	AA	AC	CC	*χ* ^2^ value (*P* value)	A	C
TP/50	0/0	7/0.1400	43/0.8600	0.28 (*P* = 0.87)	0.07	0.93
DSP/59	0/0	5/0.0850	54/0.9150	0.12 (*P* = 0.94)	0.0424	0.9576
DBP/42	8/0.1905	25/0.5952	9/0.2143	1.53 (*P* = 0.46)	0.4881	0.5119
YY/56	37/0.6611	19/0.3389	0/0	2.34 (*P* = 0.31)	0.8300	0.1700
LP54	42/0.7778	10/0.1852	2/0.0370	1.74 (*P* = 0.42)	0.8704	0.1296
DP/69	68/0.9855	1/0.0145	0/0	0.00 (*P* = 1.00)	0.9928	0.0072

*TP* represents Tibetan pigs, *DSP* diannan small-eared pigs, *DBP* dapulian black pigs, *YY*, Yorkshire pigs, *LP* landrace, *DP* duroc pig

### Association between genotype and backfat thickness in the New Huai Line

The genotypes of 117 individuals from the New Huai line were determined and the number of pigs with the AA, AC, and CC genotypes were 68, 44 and 5, respectively, and the genotype distribution conformed to Hardy-Weinberg equilibrium (Table 3). The backfat thickness of the CC genotype was significantly higher than that of the AC and AA genotypes (*P* < 0.05), and there was no significant difference between the AC and AA genotypes (*P* > 0.05).

**Table 3:** Genotype distribution of *NR1H3* exon 5-A201C and backfat thickness in New Huai line pigs

Genotype	AA	AC	CC	*χ* ^2^/*P*
Number/frequency	68/0.5811	44/0.3760	5/0.0427	0.41/0.81
Backfat thickness (mm)	11.0 ± 0.8^a^	12.2 ± 0.3^a^	13.3 ± 0.3^b^	

The different superscript letters (a, b) indicate that the means are significantly different (*P* < 0.05)

### *NR1H3* mRNA expression

**Fig. 4:**
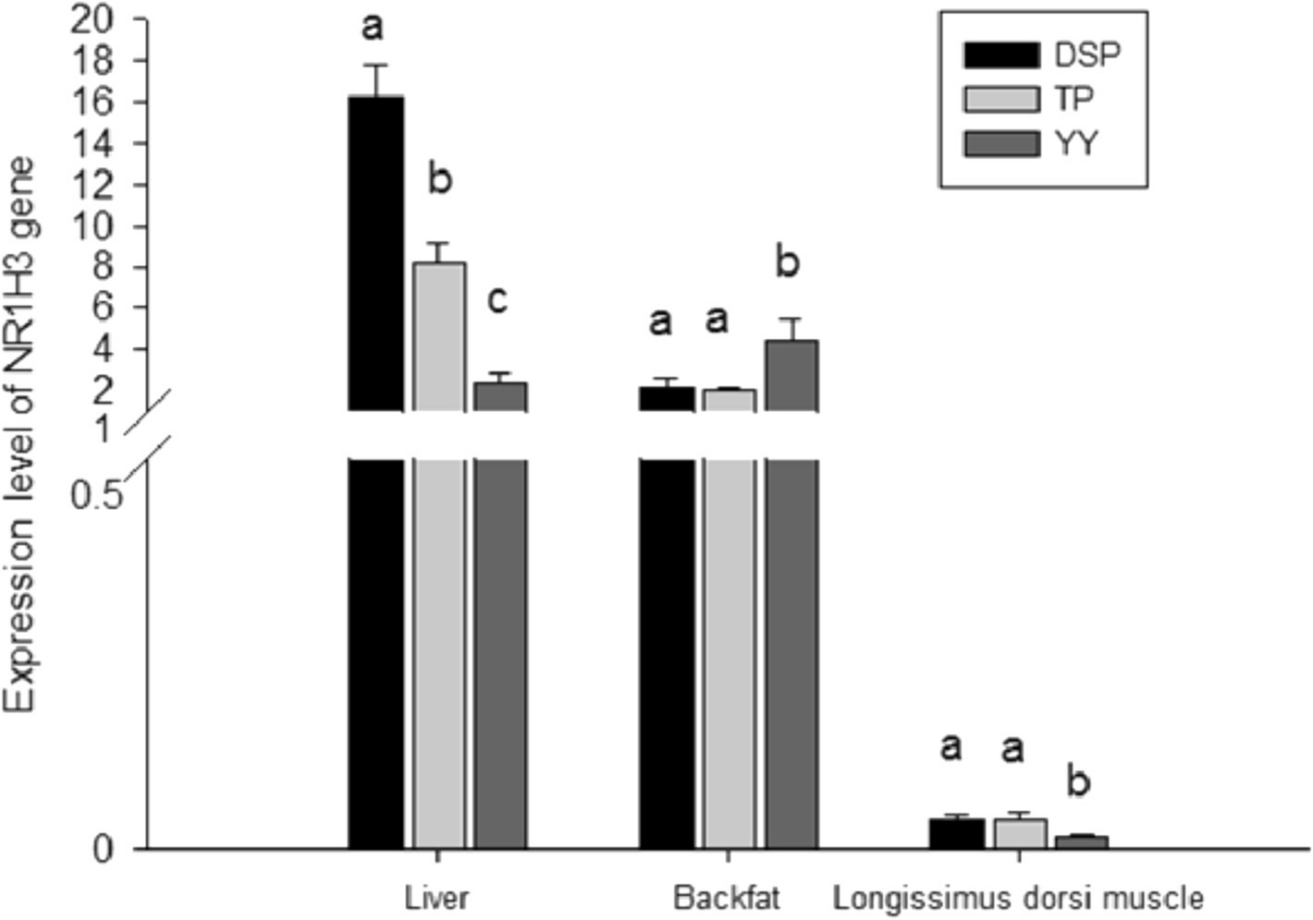
The mRNA expression level of *NR1H3* in the three tissues of three pig breeds. Each bar represents the mean ± S.E. The different letters (a, b, c) on the bars denote significant difference between breeds for the same tissue (*P* < 0.05). On the Y-axis, 0.5–1 was omitted. DSP, Diannan small-eared pig (*n* = 10); TP, Tibetan pig (*n* = 10); YY, Yorkshire pig (*n* = 10)

The *NR1H3* gene expression was maximal in the liver tissue, mid-range in the backfat, and minimal in the *longissimus dorsi* muscle of pigs (Fig. 4). The expression level in the liver of DSP was significantly higher than that of TP and YY (*P* < 0.05). Expression in the *longissimus dorsi* muscle of YY was significantly lower than that of DSP and TP (*P* < 0.05), and there was no significant difference in gene expression between DSP and TP (*P* > 0.05). In contrast, the expression level in the backfat of YY was higher than that in DSP and TP (*P* < 0.05), and no significant difference was seen between TP and DSP (*P* > 0.05) (Fig. 4).

### *NR1H3* protein expression

**Fig. 5:**
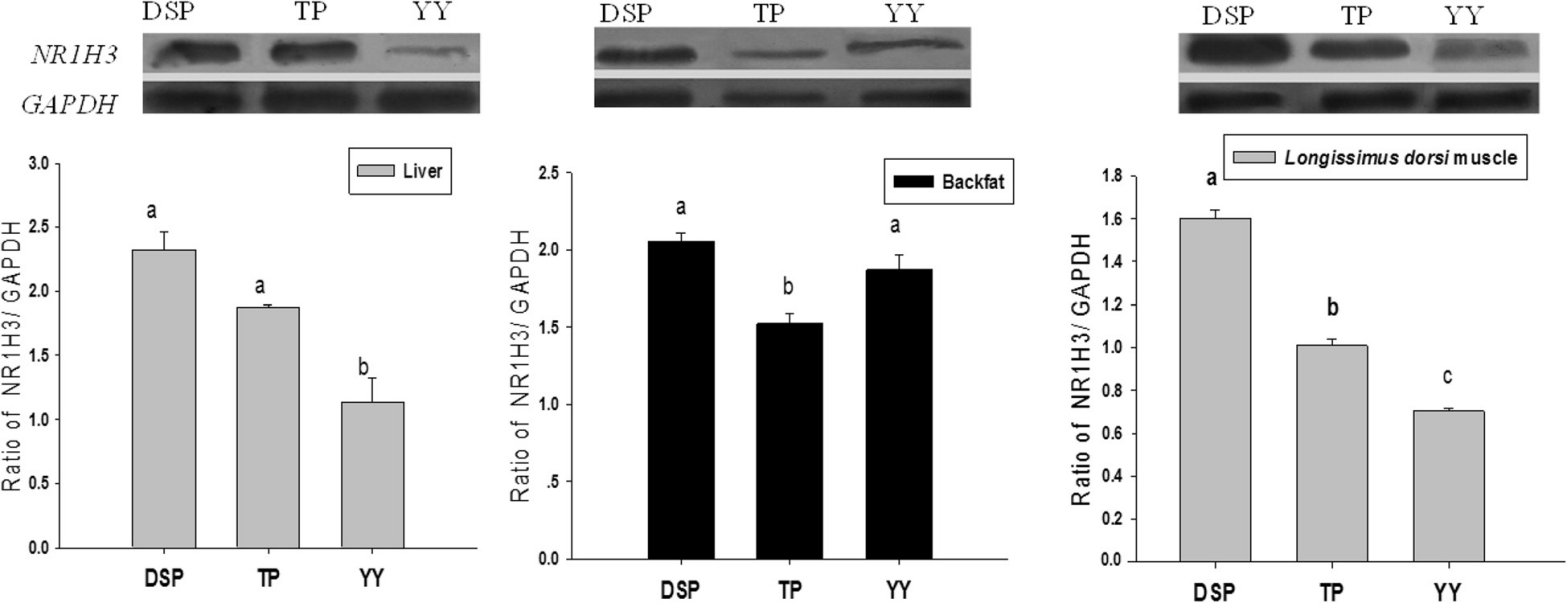
*NR1H3* protein expression level in three tissues of pigs. Each bar represents the mean ± S.E. Different letters (a, b, c) on bars denote significant differences between pig breeds for the same tissue (*P* < 0.05). DSP, Diannan small-eared pig (*n* = 10); TP, Tibetan pig (*n* = 10); YY, Yorkshire pig (*n* = 10)

*NR1H3* protein expression trends among the three examined pig breeds were similar to those for mRNA expression. In the liver and *longissimus dorsi* muscle of YY, *NR1H3* expression was significantly lower than in those of DSP and TP (*P* < 0.05) (Fig. 5). In the backfat tissue, *NR1H3* expression was lower in TP than in DSP and YY (*P* < 0.05), which is not consistent with the observed differences in mRNA expression between the breeds.

## Discussion

Fatness traits, such as backfat thickness (BFT) and intramuscular fat content (IMF), are considered economically important traits in pig breeding programs, because they influence carcass composition and meat quality [12, 13]. The rate of lipids deposited in pigs is influenced by numerous factors, including diet, age/body weight, gender, breed, environmental temperature, and body site. *NR1H3* plays important roles in lipogenesis and myogenesis [8, 9] and may affect lean muscle growth and fat content [14]. In the present study, three SNPs (exon2-C105T, exon2-G106C, and exon5-A201C) were found within the exon regions of *NR1H3*. Exon5-A201C showed interindividual variation and the frequency of the CC genotype was significantly higher in the three populations of Chinese fat-type breeds studied than in the three introduced lean-type breeds studied. The mean backfat thickness of individuals of the New Huai line with genotype CC was significantly higher than that of individuals with genotypes AC and AA (*P* < 0.05). These results suggest that the C allele of *NR1H3-*exon 5-A201C may promote lipid deposition in pigs, and increase subcutaneous fat and intramuscular fat content. *NR1H3*-exon5-A201C is a synonymous mutation that may affect gene function by altering the mRNA structure or protein folding [15, 16]. The mutation of allele A into allele C may have caused the binding transcription factor by changing the sequence from Hen-1 to Bsap. We can infer that the C allele is associated with high lipids deposition. Therefore, *NR1H3* can be used as a DNA marker for pig breeding.

*NR1H3*, a key regulatory gene of liver lipid metabolism, belongs to the orphan nuclear receptor family, and has abundant expression in the liver. In previous studies, two Chinese indigenous breeds (DSP, TP) had high backfat thickness and intramuscular fat content (IMF), whereas the Yorkshire pig, an introduced breed, had low backfat thickness and IMF [17, 18]. This indicates that DSP and TP have a distinct ability to deposit lipids. It is noteworthy that the mRNA and protein expression of *NR1H3* in the liver and *longissimus dorsi* muscles was higher in DSP and TP animals than in YY animals, which suggests that increased *NR1H3* expression may promote lipid deposition and accumulation in pigs, as it does in humans [19, 20]. An experiment on genetically obese mice demonstrated that the hepatic *NR1H3* signal induced lipogenesis and independently contributed to the development of fatty liver [21, 22]. *NR1H3* has also been linked to lipid metabolism in the liver, and it may channel fatty acids into triglyceride synthesis rather than into β-oxidation and energy production [23].

Unlike in the liver and muscle, *NR1H3* mRNA expression in the backfat tissue of YY was higher than that of DSP and TP, and the *NR1H3* protein levels in DSP and YY were higher than that in TP. This may be the result of different *NR1H3* regulatory mechanisms for lipid metabolism in the subcutaneous fat tissue and in other tissues of pigs. *NR1H3* is a potent stimulator of fatty acid and triglyceride synthesis, but it also represents a second subset of lipid-sensing receptors that are well known for their ability to sense oxysterols and regulate genes that can decrease total body cholesterol levels [24]. In the absence of *NR1H3*, the balance between fat storage and oxidation is altered, and the accumulation of hepatic cholesterol then drives the peripheral tissues to aberrantly dissipate fat-derived energy. Cholesterol is often a component of a high-fat diet and shares common metabolic pathways with lipids, and the *LXR* regulation of lipid metabolism is interdependent on its ability to maintain cholesterol homeostasis. Our results suggest that *NR1H3* promotes large amounts of fat synthesis and leads to a certain amount of fat accumulation. In order to maintain homeostasis, *NR1H3* also promotes fat oxidation, which may be the reason for the relatively high expression of *NR1H3* in the backfat tissue of YY. Endogenous hepatic *NR1H3* activity is essential for maintaining normal lipid and sterol homeostasis [25]. Understanding *NR1H3* helps in understanding the important metabolic conundrum of how to limit the accumulation of cholesterol, while simultaneously permitting the deposition of fat as an energy-rich fuel source and keeping the balance between storage and oxidation of dietary fat [26].

## Conclusions

*NR1H3*-exon-5-A201C is correlated with backfat thickness, and the expression of *NR1H3* gene may regulate lipid deposition in pigs. Further work will be necessary to investigate whether *NR1H3* plays a role in those traits involved in lean muscle fat content. Our study provides a molecular marker or functional gene that may be used for improving both the quantity and quality of pig meat production. However, further studies will be needed prior to using this marker in breeding programs. These studies should include analysis of correlations between *NR1H3* expression levels and backfat thickness and IMF content, and between SNP genotypes and lipid deposition traits.

## Abbreviations

DBP, dapulian black pig; DP, duroc pig; DSP, Diannan small-eared pig; eECL, enhanced electrochemiluminescence; *GAPDH*, glyceraldehyde-3-phosphate dehydrogenase; IMF, intramuscular fat; LP, landrace pig; LXRα, liver X receptor alpha; MAS, marker assisted selection; mRNA, messenger RNA; *NR1H3*, nuclear receptor subfamily 1, group H, member 3; PBST, phosphate buffer saline containing 0.1 % Tween 20; PCR-RFLP, polymerase chain reaction restriction fragment length polymorphism; SDS-PAGE, sodium dodecyl sulfate polyacrylamide gel electrophoresis; SNP, single nucleotide polymorphism; TP, Tibetan pig; YY, Yorkshire pig.
